# MAP kinases differentially bind and phosphorylate NOS3 via two unique NOS3 sites

**DOI:** 10.1002/2211-5463.13384

**Published:** 2022-03-15

**Authors:** Xzaviar K. V. Solone, Amber L. Caldara, Brady Wells, Hao Qiao, Lydia R. Wade, John C. Salerno, Katy A. Helms, Katherine E. R. Smith, Jonathan L. McMurry, Carol A. Chrestensen

**Affiliations:** ^1^ 12280 Department of Molecular & Cellular Biology Kennesaw State University GA USA; ^2^ 12280 Department of Chemistry & Biochemistry Kennesaw State University GA USA; ^3^ Present address: 3463 Department of Molecular Genetics and Microbiology University of Florida Gainesville FL USA; ^4^ Present address: Wake Forest Medical Center Winston‐Salem NC USA

**Keywords:** ERK2, JNK1_α1_, NOS3, optical biosensing, p38α, signaling

## Abstract

Nitric oxide synthase 3 (NOS3) is a major vasoprotective enzyme that catalyzes the conversion of l‐arginine to nitric oxide (NO) in response to a significant number of signaling pathways. Here, we provide evidence that NOS3 interactions with MAP kinases have physiological relevance. Binding interactions of NOS3 with c‐Jun N‐terminal kinase (JNK1_α1_), p38α, and ERK2 were characterized using optical biosensing with full‐length NOS3 and NOS3 specific peptides and phosphopeptides. Like p38α and ERK2, JNK1_α1_ exhibited high‐affinity binding to full‐length NOS3 (*K*_D_ 15 nm). Rate constants exhibited fast‐on, slow‐off binding (*k*_on_ = 4106 m ^−1^s^−1^; *k*_off_ = 6.2 × 10^‐5^ s^−1^). Further analysis using synthetic NOS3 peptides revealed two MAP kinase binding sites unique to NOS3. p38α evinced similar affinity with both NOS3 binding sites. For ERK2 and JNK1_α1,_ the affinity at the two sites differed. However, NOS3 peptides with a phosphate at either S114 or S633 did not meaningfully interact with the kinases. Immunoblotting revealed that each kinase phosphorylated NOS3 with a unique pattern. JNK1_α1_ predominantly phosphorylated NOS3 at S114, ERK2 at S600, and p38α phosphorylated both residues. *In vitro* production of NO was unchanged by phosphorylation at these sites. In human microvascular endothelial cells, endogenous interactions of all the MAP kinases with NOS3 were captured using proximity ligation assay in resting cells. Our results underscore the importance of MAP kinase interactions, identifying two unique NOS3 interaction sites with potential for modulation by MAP kinase phosphorylation (S114) and other signaling inputs, like protein kinase A (S633).

## Graphical abstract

Nitric oxide synthase 3 (NOS3, eNOS) is a major vasoprotective enzyme that binds MAP kinases in cells. Representative MAP kinases (JNK1_α1_, p38α, and ERK2) bound NOS3 with high affinity at two hypothesized binding motifs and each kinase uniquely phosphorylated NOS3. Phosphorylation adjacent to the motifs disrupted MAP kinase binding, suggesting NOS3 phosphorylation status could modulate these interactions.

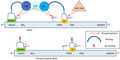

**Abbreviations**
AI: autoinhibitory loop
BLI: biolayer interferometry
CaM: calmodulin
CDK: cyclin dependent kinase
eNOS: endothelial nitric oxide synthase
ERK2: mitogen‐activated protein kinase 1
GST: glutathione S‐transferase
HMEC‐1: human microvascular endothelial cells
JNK1_α1_
: mitogen‐activated protein kinase 8
MAP kinase: mitogen‐activated protein kinase
metHb: methemoglobin
NO: nitric oxide
NOS1: nitric oxide synthase 1, also known as neuronal (n)NOS
NOS2: nitric oxide synthase 2, also known as inducible (i)NOS
NOS3: nitric oxide synthase 3, also known as endothelial (e)NOS
NtMAP: N‐terminal MAP
oxyHb: oxyhemoglobin
p38α: mitogen‐activated protein kinase 14
PKA: cAMP‐dependent protein kinase
TCSPC FLT: time‐correlated single‐photon counting fluorescence lifetime spectrometry

Nitric oxide synthase 3 (NOS3), also known as eNOS, is a major vasoprotective enzyme that catalyzes the conversion of l‐arginine to nitric oxide (NO) in response to a significant number of signaling pathways. Endogenous NO produced in the right location at the proper concentration influences angiogenesis and controls vascular tone, while dysregulation can lead to disease [1, 2, 3, 4]. When replete with calcium, calmodulin (CaM) directly binds and activates NOS3. Posttranslational modifications, including phosphorylation, acetylation, and glutathionylation, further regulate NOS3. Recent results underscore the complexity of NOS3 regulation by phosphorylation, showing that phosphorylation of a well‐documented activating site (S1177, human NOS3 numbering used throughout) does not necessarily correlate with increased synthesis of NO *in vivo* [5]. Much less is understood about the fate of multiply modified NOS3. Some modifications like T495 phosphorylation block interaction with CaM and would be expected to trump the effects of other modifications [7].

Mitogen‐activated protein (MAP) kinases represent important nodes in signaling networks that respond to a variety of stimuli [8]. There are several family members of MAP kinases including extracellular signal‐regulated kinases (ERK), p38, and c‐Jun amino‐terminal kinase (JNK, also known as stress‐activated protein kinase, SAPK). Although all MAP kinases participate in intracellular communication, they differ in which pathways activate them, how they are regulated, and the specific cellular outcomes they produce. Despite these differences, three family members (ERK, p38, and JNK) have been shown to interact with and phosphorylate NOS3 [2, 4, 6, 9, 10, 11, 12].

MAP kinases have two known recruitment sites for interactions with proteins, including activators, substrates, and phosphatases. The literature around these motifs is significant (reviewed [8, 13, 14]). Herein, we use the terminology of the D (2 basic residues, 2‐6 amino acids, followed by a hydrophobic residue) and DEF motifs (FXFP). ERK and p38 can interact with proteins independently through these distinct motifs and some proteins interact through both [15, 16, 17]. The D motif has been observed to recruit protein interactions in both orientations, ‘forward’ N to C terminus or ‘reverse’ C to N terminus [16]. Previously, we reported nanomolar affinity of p38α and ERK2 binding to NOS3 but not NOS1 and hypothesized the autoinhibitory loop (AI) as the likely site of interaction because of the pentabasic sequence found only in NOS3 [4]. The AI site is likely a reverse D motif utilizing L622 and/or V623 as the hydrophobic residue(s).

JNK binds and phosphorylates NOS3 in endothelial cells; inhibition and dominant negative experiments showed that JNK2 inhibited cellular NO production [10] through phosphorylation at the S114 site. They hypothesized a JNK binding motif (^96^PRRCLGSLVLP^106^) just upstream (N‐terminal) of the S114 site [10]; like the AI motif, this region is also unique to NOS3 (Fig. 1). We utilized a NOS3 peptide that encompasses the above motif and includes the downstream S114 phosphorylation site, named the N‐terminal MAP (NtMAP) site, in parallel with a peptide that encompassed the AI reverse D motif to determine the ability of these NOS3 regions to bind MAP kinases.

**Fig. 1:**
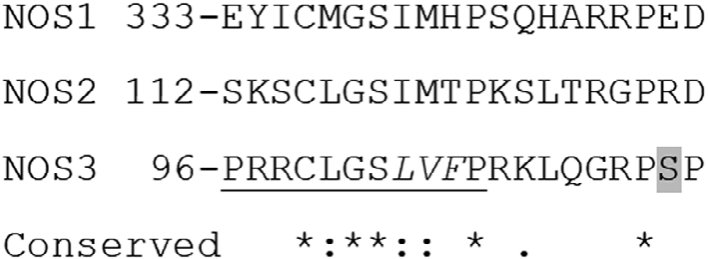
The proposed N‐terminal MAP kinase (NtMAP) site is unique to NOS3. Alignment of the proposed NtMAP kinase binding site in human NOS3, to human NOS1 and NOS2 using T‐Coffee [27, 28]. The alignment of this subsection of NOS3 from 96‐115 to NOS1 and 2 is identical when aligned with all three full sequences or just when the subsequence is used against the full sequences of NOS1 and NOS2. The postalignment analysis of the sequences above was ‘good’ for all residues with the shown conservation [29]. The underlined segment is the interaction site proposed previously for JNK [10]. The MAP kinase interacting motif LVF (italics above)—ΦXΦ motif is minimally conserved in NOS1 and NOS2 providing evidence that it might also be a unique NOS3 interaction site. The unique NOS3 SP site is highlighted in gray. * ‐ exact conservation of amino acids, : ‐ conservation of amino acids with highly similar properties,. ‐ conservation of amino acids with weakly similar properties.

Prior to our examination of ERK2 and p38α interactions with NOS3, ERK1/2 interactions with NOS3 in bovine aortic endothelial cells were shown to be modulated in response to bradykinin [9]. Later work showed that phosphorylation at S114 is inhibitory through indirect methods, including binding of peptidyl‐prolyl cis‐trans isomerase NIMA‐interacting 1 (Pin1) and prevention of c‐Src binding and phosphorylation of NOS3 at Y83 [18, 19]. Others have shown cellular interactions between NOS3 and p38 [20]. The affinity of interaction between JNK and NOS3 has not been reported previously.

Evidence continues to strengthen around the hypothesis that NOS3 interactions with MAP kinases have physiological relevance. Herein, we add to that body of work, with *in vitro* interaction and activity data. Our current analysis *in vitro* shows NOS3 is differentially phosphorylated by ERK2, p38α, and JNK1_α1_. JNK1_α1_‐ and p38α‐mediated phosphorylation of S114 was easily detected when compared to ERK2, while p38α and ERK2 phosphorylation of S600 was robust when compared to JNK1_α1_. Activity analysis shows that *in vitro* none of these modifications altered NO production. Our *in vitro* binding data show that JNK1_α1_ binds NOS3 with nanomolar affinity. We used peptides and phosphopeptides to test the two hypothesized MAP kinase interaction motifs on NOS3, the N‐terminal MAP kinase (NtMAP) and the reverse D motif in the autoinhibitory loop (AI) [4, 10]. We found that when phosphates are present at certain residues both regions of NOS3 lose the ability to bind MAP kinases, much as phosphorylation of T495 inhibits CaM binding [2, 7].

## Materials and methods

### Materials

Standard reagents were purchased from Sigma‐Aldrich, Bio‐Rad, Fisher, and Genesee Scientific. 5‐Aminolevulinic acid was from Cayman Chemical. Protease Arrest was from G‐Biosciences. Monoclonal antibody against NOS3 (#610297) was from BD Biosciences. Phospho‐specific S600/S602 NOS3 antibody (0.9 mg·mL^−1^ stock) was described previously [6] and can be purchased from Millipore Sigma (ABS1631). Phospho‐specific Ser114/S116 NOS3 antibody (Lot# 2973090) was also from Millipore. cAMP‐dependent Protein Kinase (PKA), Catalytic Subunit (lot #P6000L) was from New England Biolabs. GST‐purified MAP kinases ERK2 (Lot #P1662‐5) and JNK1 (Lot #X561‐2) were from SignalChem. In later experiments, His‐purified MAP kinases ERK2 & p38α (ERK2—addgene #39212, and p38α [21]) and GST‐JNK1a1 (addgene #47574 and with the active upstream MKK #47580) were made in house (both inactive and active), as described previously for ERK2 [22], p38α [21], and for JNK1a1 adapted from [23].

### Purification of NOSs bovine and human

Initially, NOS3 and NOS1 (bovine) were heterologously expressed in E. coli BL21(DE3), purified and assayed for activity as described [24, 25]. Later experiments utilized a second NOS3 (human) expression system, polyHis‐pet19b.NOS3 vector (Ampicillin resistant) codon‐optimized for translation in *E. coli*. Hi‐Control competent cells were transfected with chaperones CPN 10 and 60 from the Artic Express strains (Agilent Technology, Santa Clara, CA, USA) and the polyHis‐pet19b.eNOS, plated on ampicillin and gentamicin. Ten percent of a 50‐ml overnight culture was used to inoculate 1 L of terrific broth with ampicillin, riboflavin (3 µm), and aminolevulinic acid (1 mm) in 2.8 L fernbach flasks. Flasks were grown shaking (220 r.p.m.) in the dark at 37 °C. At an OD600 of 0.8–1.0 (4–5 h), cultures were supplemented with adenosine‐5‐triphosphate (ATP, 0.1 mg·mL^−1^) and induced with isopropyl‐beta‐D‐thiogalactopyranoside (IPTG, 1 mm). The cells were incubated for 16–48 h at 23 °C (220 r.p.m.) again in the dark. Bacteria were harvested and stored as pellets at −80 °C. All steps of the purification were performed at 4 °C. Typically, two pellets were resuspended at 4 °C in purification buffer (50 mm Tris/HCL (pH 8), 0.5 m NaCl, 10 mm imidazole, 10% glycerol), containing 0.5% Tween‐20, 6 mm BME, ~ 7 micrograms of DNase I, 2× protease arrest 100 µm BH_4_, 5 µm FAD, and 5 µm FMN, then lysed 2× using a French press (20 000 psi). Lysates were centrifuged ~ 27 000× ***g*** for 20 min to pellet unbroken cells and cell debris. The supernatants were incubated with 1 mL of TALON cobalt resin beads pre‐equilibrated with purification buffer. The beads were washed three times using purification buffer plus 1 mm BME, 5 µm FAD, and 5 µm FMN. NOS3 protein was eluted with purification buffer containing 1 mm BME and 250 mm imidazole in 1 mL fractions. Fractions with higher protein concentrations were dialyzed overnight using a Slide‐A‐Lyzer dialysis cassette in NOS buffer (10 mm HEPES (pH 7.4), 150 mm NaCl, 0.05% Tween‐20, 10% glycerol and 1 mm DTT). Purified NOS3 was stored at −80 °C. Protein homogeneity was confirmed using SDS/PAGE with Coomassie blue staining, and purified protein was subjected to immunoblotting with anti‐NOS3 antibody. The final protein concentrations were measured in each fraction by total protein concentration and by heme concentration. Concentrations of heme‐containing NOS3 were determined from the absorption spectrum of the protein using the extinction coefficient ε_400_ = 100 mm ^−1^ cm^−1^ for the ferric enzyme [26].

### Purification of kinases

Kinases were produced in both the inactive (nonphosphorylated) and active (phosphorylated) forms, using *E*. *coli* expressing the kinase alone or using a system that coexpresses the upstream activating kinase in a constitutively active form, generally as directed by prior work [21, 22, 23]. Plasmids were freshly transformed or cotransformed into BL21(DE3) pLysS. Overnight cultures grown from single colonies were subcultured into 1‐L Luria‐Bertani broth containing the required antibiotics depending on the kinase or kinases and grown with shaking at 37 °C for 8 h. Cells were induced with 1 mm IPTG and growth continued for 16 h at 16 °C. Cells were harvested by centrifugation at 10 000× ***g*** and frozen at −80 °C.

Purification was performed via immobilized metal affinity chromatography or with Glutathione Sepharose 4B beads (GE). Briefly, cell pellets were thawed on ice and resuspended in PBS supplemented with 5 mm DTT. 1 mg·mL^−1^ DNAse, 0.25 mg·mL^−1^ lysozyme, and 1× Halt protease inhibitor cocktail (Thermo Scientific) were added during resuspension. Cells were lysed and cleared as above. Clarified lysate was incubated in the appropriate resin equilibrated in PBS for 1 h at 4 °C and washed 3× with PBS. His tagged proteins were eluted in imidazole, and GST tagged proteins were eluted in 50 mm Tris with 10 mm glutathione, pH 8. Protein containing fractions were pooled, concentrated, and buffer exchanged by passage over a Zeba spin desalting column (Thermo Scientific, Waltham, MA, USA) into PBS with 1 mm DTT and 2 mm EDTA.

### Optical biosensing

Biolayer interferometry (BLI) experiments were performed on a FortéBio (Menlo Park, CA) Octet QK biosensor using streptavidin (SA) sensors. Assays were performed in opaque 96‐well microplates at 25 °C. All volumes were 200 μL. Active GST‐JNK1_α1_ (SignalChem) was biotinylated by amine crosslinking to NHS‐LC‐LC‐biotin (succinimidyl‐6‐[biotinamido]‐6‐hexanamidohexanoate) (Thermo Fisher, Waltham, MA, USA). Reactions were performed at a 5 : 1 molar ratio of biotin to protein for 30 min at room temperature followed by separation of protein from free biotin by passage over a desalting column. Biotinylated JNK1_α1_ was loaded onto sensors for 300 s, which was sufficient to saturate them. Baseline was established by dipping sensors into binding buffer (50 mm Tris, pH 7.4, 10 mm NaCl, 10% glycerol, 0.01% Tween‐20) prior to moving sensors to wells containing varying concentrations of serially diluted NOS3 in the same buffer. Association was measured for 300 s, after which sensors were moved to wells containing buffer only and dissociation was monitored for another 300 s. Raw data were normalized for baseline drift by subtracting signal from a ligand‐saturated sensor exposed to buffer only throughout the experiment and then iteratively fit to a one‐state global association‐then‐dissociation binding model using graphpad Prism. Total binding was used for analysis as nonspecific binding measured by exposing sensors without ligand to analyte was negligible at all NOS3 concentrations used; that is, total binding was effectively specific binding due to very low nonspecific binding.

For peptide binding experiments, N‐terminally biotinylated peptides (Biomatik) (Table 1) in PBS with 10% glycerol, 0.005% tween, and 1 mm DTT were loaded onto SA sensors as above. After establishing baseline, sensors were exposed to serial dilutions of analyte MAP kinases ranging from 31 to 1000 nm. Peptide experiments were performed using parameters identical to the protein ligand experiments excepting the buffer was PBS with 10% glycerol, 0.005% tween, and 1 mm DTT throughout.

**Table 1:** Peptides and phosphopeptides designed to test the hypothesized MAP kinase binding sites. The location and name of the peptide is in the left column and the sequence is in the column to the right. Phosphorylated residues denoted with an *. Human numbering and sequences were used, for the NtMAP sequence human contains R at 97 and F at 105, bovine has C and L, respectively, at the aligned residues.

NOS3 location	Peptide sequence
NtMAP	CTPRRCLGSLVFPRKLQGRPSPGPPA
NtMAP pS114	CTPRRCLGSLVFPRKLQGRPS*PGPPA
AI	YKIRFNSISCSDPLVSSWRRKRKESSNTDSA
AI pS615	YKIRFNS*ISCSDPLVSSWRRKRKESSNTDSA
AI pS633	YKIRFNSISCSDPLVSSWRRKRKES*SNTDSA

Goodness‐of‐fit parameters for all sensorgram fits can be found in Supplemental Information (Table S1).

### Alignment

Full‐length human NOS1 (9NP_000611.1), NOS2 (NP_000616.3), and NOS3 (NP_000594.2) protein sequences were aligned using T‐COFFEE [27, 28]; then, full sequences of NOS1 and NOS2 were aligned with NOS3 subsequence 96‐115, which encompasses the examined NtMAP kinase site and the S114 phosphorylation site. T‐COFFEE provides the resulting alignment with indications of conservation at each residue, BAD, AVG, and GOOD. The resulting alignment of the subsequences shown in Fig. 1 was analyzed using the TSC evaluation [29].

### *In Vitro* kinase assay with NOS3

Active (HIS‐NOS3) was incubated alone or with individual kinases (ERK2, p38α, JNK1_α1_, and PKA) in 100 µL reaction buffer (20 mm HEPES, 0.5 mm DTT, 5 mm MgCl_2_, 0.5 mm ATP, and 10% glycerol at a pH of 7.4). The reactions were performed at 22‐25 °C for up to 1 h, and for the examination of nitric oxide activity, samples were incubated on ice for the immediate detection of NO via the oxyhemoglobin to methemoglobin assay (see below). For immunoblotting analysis to examine phosphorylation, kinase samples were quenched with 2× sample buffer spiked with 25 mm DTT and heated to 95 °C for 5 min. Boiled samples (at equal volumes) were electrophoresed and transferred to PDVF for immunoblot staining.

### Oxyhemoglobin assay

The oxyhemoglobin assay (oxyHb assay) is a spectroscopic method that measures the oxidative reaction of oxyhemoglobin at 421 nm (oxyHb) and methemoglobin (metHb) at 401 nm [26]. Kinase assay reactions quenched on ice were used to measure NO synthesis and consumption. All oxyHb reactions were performed at 25 °C in a BioTek 96‐well UV‐visible spectrophotometer. Each reaction (100 µL total volume) was mixed with 40 µL of reaction buffer (1 μm FAD, 1 μm FMN, 10% glycerol, 12 nm BH4, 100 μm l‐arginine, 10 mm Tris/HCl, pH 7.5 at 25°, 250 μm CaCl_2_, 0.20 mg·mL^−1^ BSA, 0.31 mg Hemoglobin, 7–10 U Superoxide Dismutase, 7–10 U Catalase, and 17 μg Calmodulin) and up to 40 µL (6–10 µg) of phosphorylated NOS3. Absorbance at 401 nm and 421 nm were read for 1 m at 15‐s intervals. To initiate the reaction, 20 µL of 200 mm NADPH reconstituted in Tris/HCl (1.4 m pH 7.5 at room temperature) was added. Measurements were recorded for 10 min at 15‐s intervals.

### NOS3 activity calculation

To calculate NOS3 activity, the change in absorbance/unit time was divided by the extinction coefficient (ε_401‐421_ = 77 200 m ^−1^cm^−1^ (ε_401_ = 38 600 m ^−1^cm^−1^)) [26]. This yielded NOS3 activity in units of molar change/unit time. Activity of kinase exposed NOS3 was compared to the NOS3 control activity (comparably treated NOS3 but not phosphorylated), and the data are presented as a percent of the control NOS3.

### Immunoblotting

All samples were loaded at equal volumes on 3–8% gradient tris‐acetate gels. All gels were transferred to PVDF membranes and transferred for either 2 h at 40V or for 20V overnight. Membranes were blocked with one block (Li‐Cor Biosciences) for 30 m and incubated with respective antibodies (at manufacturers recommend concentrations for immunoblotting, 1 : 500 for the anti‐pS600‐NOS3) overnight at 4 °C. Blots were brought to RT for 30 m and washed 3× with 1X TBST for 10 m, probed with a fluorometric secondary antibody (Li‐Cor IRDye®) for 1 h, and washed before visualizing with a Li‐Cor Odyssey.

### Cell lines and culture

Human microvascular endothelial cells (HMEC‐1) (CRL‐3243, ATCC) were cultured in MCDB131 medium. HMEC‐1 cells were maintained as described by the ATCC in MCDB131 supplemented with 10% FBS, 1 µg·mL^−1^ Hydrocortisone, 10 mm Glutamine, and 10 ng·mL^−1^ Epidermal Growth Factor under an atmosphere of 5% CO2 at 37 °C.

### Proximity ligation assay (PLA)

PLA was performed using the Duolink *In* *Situ* Red Kit (Sigma‐Aldrich, DUO92101) as directed by the manufacturer. HMEC‐1 cells grown on slides were treated as directed to fix and then primary antibodies were incubated on the cells for 16 h at 4 °C at the following concentrations: ERK1/2 (Cell Signaling Technologies (CST), 9102; Danvers, MA, USA) 1 : 100, p38 MAPK (D13E1) (CST, 8690) 1 : 200, NOS3 (BD Biosciences, 310297) 1 : 100, and JNK1,2,3 (Bioss, bs‐2592R) 1 : 50. DAPI (10 µg·mL^−1^) was added for 20 min at room temperature before the final PLA washes. The slides were then mounted with Vectashield antifade mounting medium (Vector Laboratories, H‐1000) and allowed to dry before imaging on an inverted Zeiss LSM700 confocal microscope with a 40× EC Plan‐Neofluar objective.

## Results

### JNK1_α1_ binds to NOS3 with high affinity in a concentration‐dependent manner

**Fig. 2:**
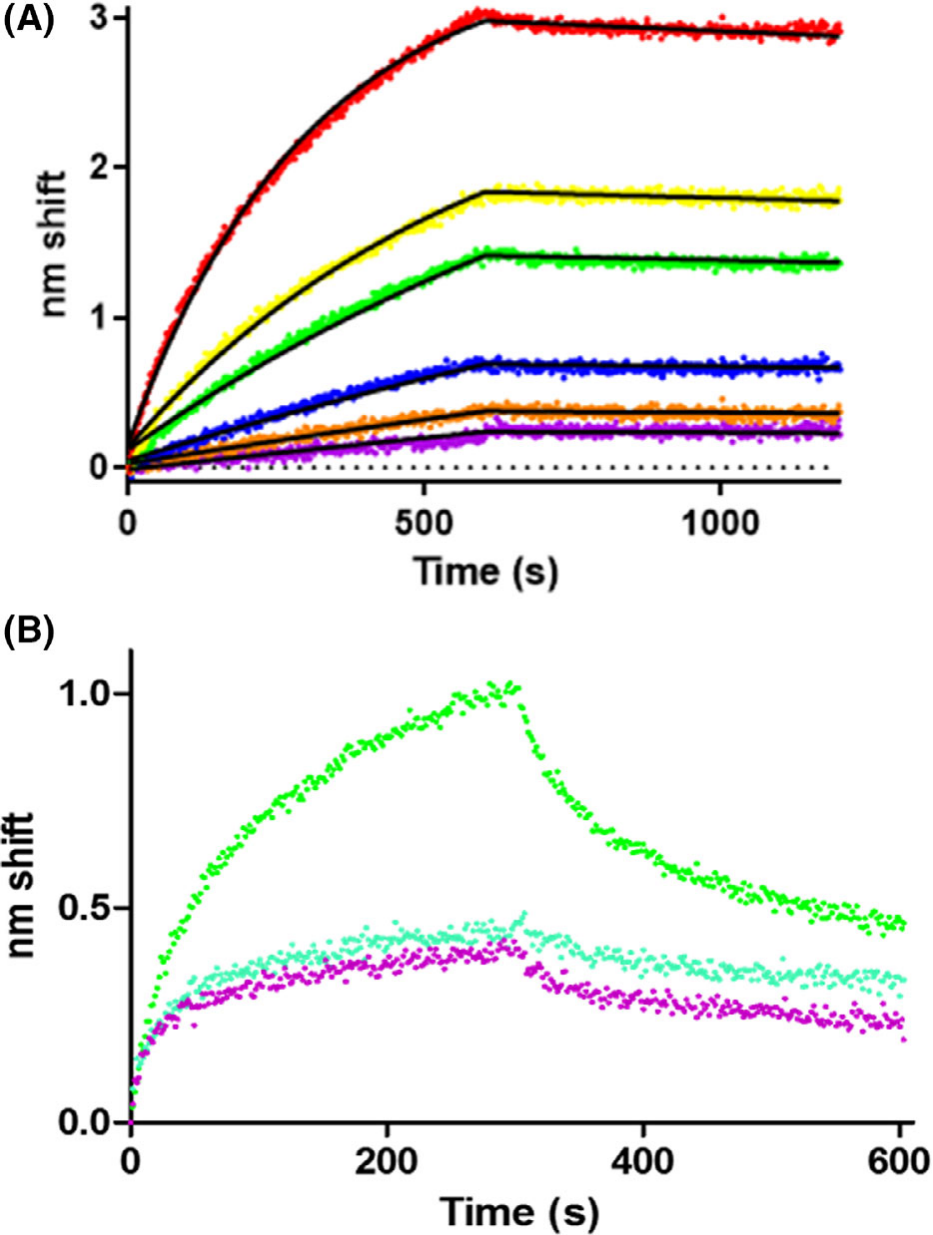
JNK1_α1_ binds to NOS3 in a concentration‐dependent manner. (A) BLI sensorgram of JNK1_α1_ binding to NOS3. Purified NOS3 at concentrations ranging from 25 to 800 nm (purple, 25 nm; orange, 50 nm; blue, 100 nm; green, 200 nm; yellow, 400 nm; red, 800 nm) were assayed for binding to ligand GST‐JNK1_α1_. Raw data are shown with fits to a one‐state association‐then‐dissociation model. The association phase was from 0 to 600 s, whereas dissociation was from 600‐1200s. (B) No evidence of JNK1_α1_ binding to NOS1 beyond nonspecific binding. Green denotes response to ligand calmodulin, blue represents response to ligand JNK1_α1_ and purple is no ligand (NS) control.

Active JNK1_α1_ binds NOS3 (bovine) with high affinity as demonstrated with biolayer interferometry (BLI), an optical biosensing technique capable of determining kinetic and affinity constants in real‐time (Fig. 2) [30]. A global fit to a one‐state model of an association‐then‐dissociation experiment at five different analyte concentrations produced a *k*_on_ of 4106 m ^−1^s^−1^ and a *k*_off_ of 6.2 × 10^−5^ s^−1^, yielding an affinity constant of 15 nm. Goodness‐of‐fit measures demonstrate the veracity of analysis; 95% confidence intervals were 4046‐4165 m ^−1^s^−1^ for *k*_on_ and 5.7 × 10^‐5‐4^‐6.6 × 10^‐5^ s^−1^ for *k*_off_. Eliminating the 400 nm trace resulted in negligible improvement in goodness of fit and a small change in *K*_D_, to 18 nm. Binding to sensors coated with the fusion partner without JNK was negligible as was binding to sensors without ligand.

We previously reported similar binding affinities of p38α and ERK2 to NOS3 with a *K*_D_ of 80 nm and 160 nm, respectively [4]. When biotinylated NOS1 was immobilized on a streptavidin sensor and immersed in purified active JNK1_α1_ no binding could be detected (Fig. 2B). While there is substantial nonspecific binding inherent in the NOS1 sample, it is quite clear that there is no observable specific binding. This result parallels previous studies in which p38α and ERK2 also evinced no binding to NOS1 [4]. The NOS1 isoform lacks the pentabasic sequence in the autoinhibitory loop that we hypothesized to be the binding site for MAPKs [4]. The proposed NtMAP binding site (^96^PRRCLGSLVFPRK^109^) is also not conserved in NOS1 or NOS2 (Fig. 1).

### MAP kinases phosphorylate NOS3 uniquely while NO production is unchanged

**Fig. 3:**
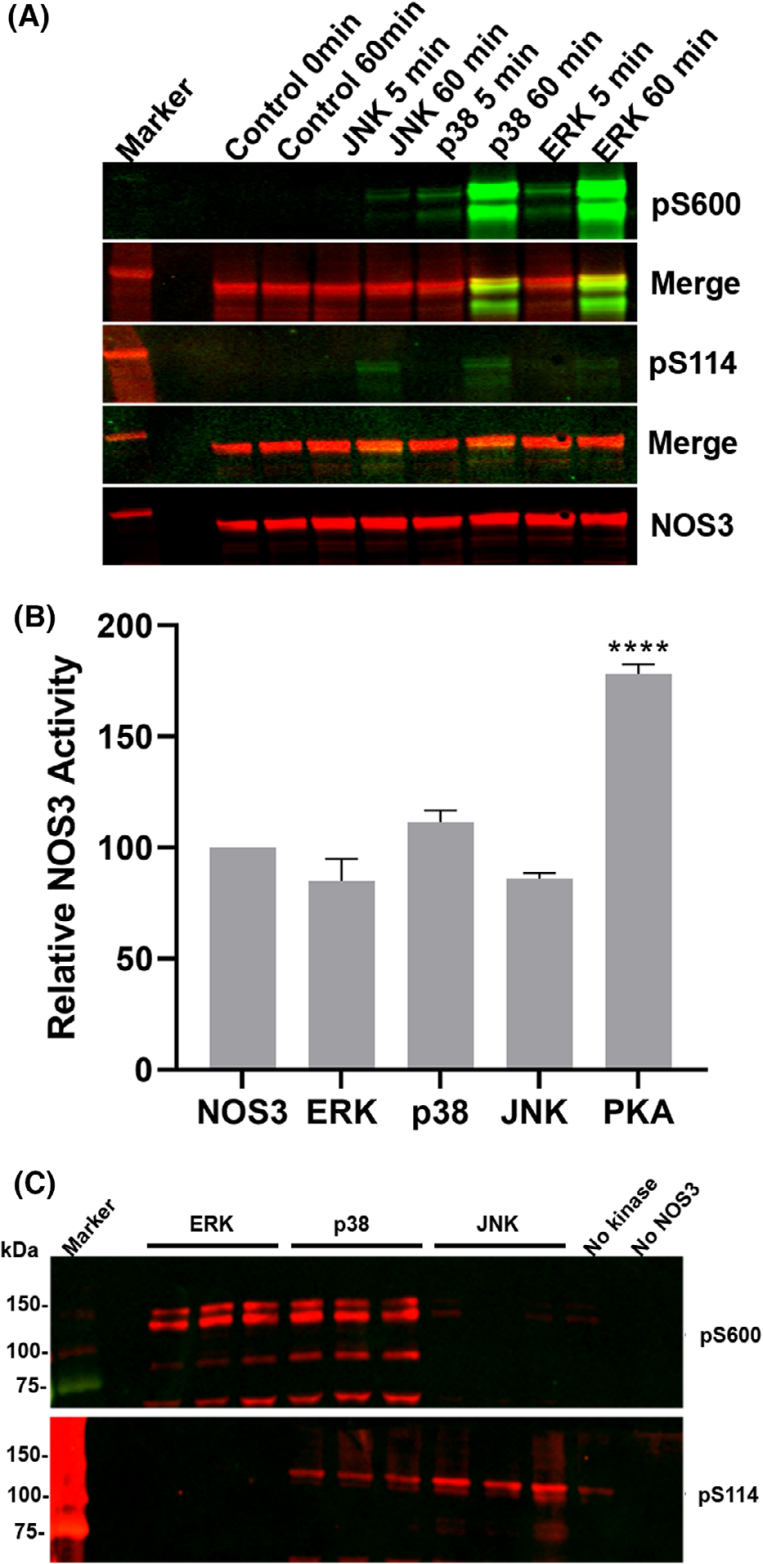
JNK1_α1_, p38α, and ERK2 phosphorylate NOS3 at different sites but do not significantly change NO production. (A) Active kinases, JNK1_α1_ (0.16 µg), p38α (0.68 µg), or ERK2 (0.16 µg), were incubated in kinase reactions with NOS3 (150 µg), samples were taken at 5 and 60 min and analyzed by immunoblot using phospho‐specific antibodies S600 [6] and S114 green (Millipore) and anti‐NOS3 red (BD Biosciences), yellow indicates both signals. (B) NOS3 (2.5 µm) was incubated with either ERK2 (14 nm) *N* = 10, p38α (14 nm) *N* = 4, JNK1_α1_‐GST (63 nm) *N* = 3, or PKA (14 nm) *N* = 4, for 30 min in kinase reactions were used to measure the change in oxyhemoglobin (401 nm) to methemoglobin (421 nm) in triplicate for 30 min. Approximately 8 µg of NOS3 from the kinase reaction was used in each of the OxyHb. One‐way ANOVA test was used to calculate the *P* values (****P < 0.001). The data are expressed as mean ± SD. (C) Immunoblot analysis of NOS3 from one of the OxyHb assays in triplicate.

Treatment of NOS3 *in vitro* with each MAP kinase followed by immunoblotting revealed that JNK1_α1_ phosphorylates S114, ERK2 phosphorylates S600 and p38α phosphorylates both residues (Fig. 3A). Probing with anti‐NOS3 yielded a species commensurate with the monomeric molecular weight (~ 150 KD) that is cross‐reactive with the phosphorylated signals. Thus, the different MAP kinases show unique patterns of NOS3 phosphorylation.

Portions of the NOS3 phosphorylation assays were analyzed for nitric oxide production, using PKA as a positive control. As expected, PKA‐mediated phosphorylation increased production of NO; despite their unique phosphorylation patterns, none of the MAP kinase significantly altered NO production compared to untreated NOS3 (Fig. 3B). Portions of the OxyHb reactions were subjected to immunoblot analysis (Fig. 3C) and showed the same patterns of phosphorylation observed in Fig. 3A. It is important to note that we cannot interpret the degree of NOS3 phosphorylation, just that NOS3 was phosphorylated in a concentration (data not shown) and time‐dependent manner. The ERK findings shown here disagree with our previous report [6], in which we found approximately 50% inhibition of NOS3 activity. Several differences give us high confidence in our more recent results. Most importantly, here we used a different source of ERK2, thus also eliminating contaminating glutathione (a known inhibitor of NOS [31]); here, we also use the more reliable OxyHb assay and human rather than bovine NOS3.

### MAP kinases bind uniquely with unphosphorylated but not phosphorylated NOS3 peptides

**Fig. 4:**
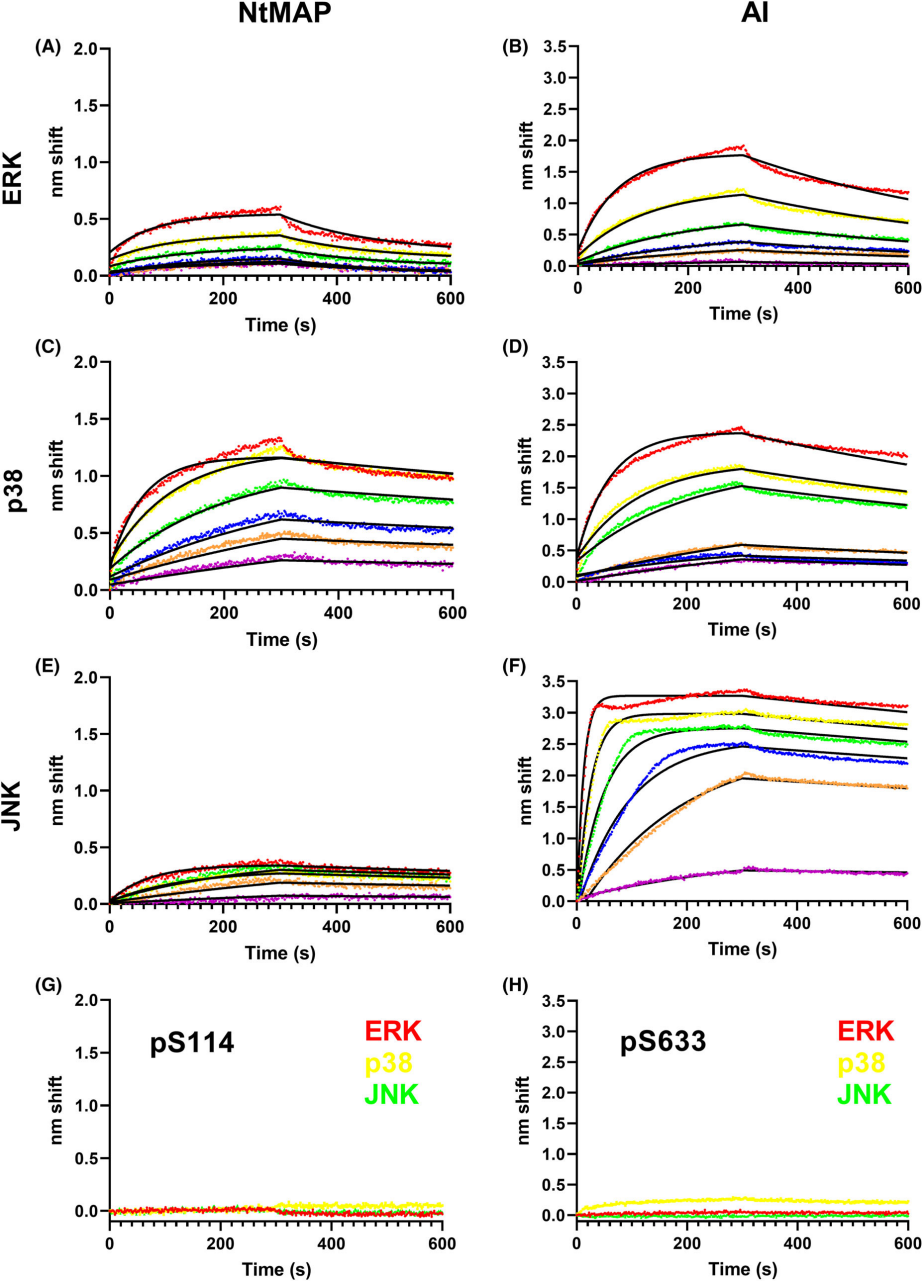
MAP kinases bind unphosphorylated but not phosphorylated NtMAP and AI peptides. BLI sensorgrams of analyte MAP kinases binding to ligand peptides. Ligand–analyte pairs are (A) NtMAP‐ERK2, (B) AI‐ERK2, (C) NtMAP‐p38α, (D) AI‐p38α, (E) NtMAP‐JNK1_α1_, (F) AI‐JNK1_α1_. Analyte concentrations in A‐F were 1000 nm (red), 500 nm (yellow), 250 nm (green), 125 nm (blue), 63 nm (orange), and 31 nm (purple). Raw data are rendered as points with fits to a global one‐state association‐then‐dissociation model shown in black. (G), Ligand NtMAP with phosphorylated S114 and H), Ligand AI with phosphorylated S633 evinced negligible binding to kinase analytes ERK2 (red), p38α (yellow), and JNK1_α1_ (green), indicating very low or no affinity.

To better understand how the kinases interact with NOS3, we used BLI to measure binding affinity (rather than kinase activity) of the inactive (unphosphorylated) MAP kinases with synthetic biotinylated NOS3 peptides. We tested both hypothesized interaction sites and the impact of nearby phosphorylation sites using: (a) a nonphosphorylated peptide corresponding to the putative NtMAP site (Fig. 1); (b) the identical peptide with S114 phosphorylated; (c) a nonphosphorylated peptide corresponding to the AI loop site [6]; and (d) an identical peptide phosphorylated at the position corresponding to S633. The resultant sensorgrams are shown in Fig. 4 fit to global one‐state binding models to determine binding parameters. While there are complexities present in the data (see Discussion), a one‐state models fit very well to all peptide‐kinase curves. Kinetic and affinity constants are listed in Table 2. All three kinases bound both unphosphorylated peptides with submicromolar affinity that in most cases approximated the affinity observed for full‐length NOS3 reported above and in our prior report [4]. Interestingly, all three kinases bound with higher affinity to the AI peptide than the NtMAP peptide, but JNK1_α1_ had a higher affinity (≥10 fold) for both peptides compared to ERK2. p38α bound with nearly identical affinity to each peptide and had stronger affinity for both peptides than ERK2. The presence of a phosphate at S114 or S633 effectively blocked interaction with all kinases (Fig. 4G & H).

**Table 2:** Kinetic and affinity constants for NOS3 peptide binding to MAP kinases determined by BLI. Parameters were determined by fitting to a global one‐state binding model. nd, not determined.

Ligand	Kinase	*K*_D_ (nm)	*k*_on_ (m ^−1^ s^−1^)	*k*_off_ (s^−1^)
NtMAP	ERK	940	6.6 × 10^3^	6.2 × 10^‐2^
	p38	31	1.7 × 10^4^	5.3 × 10^−4^
	JNK	38	1.4 × 10^4^	5.4 × 10^−4^
p114	ERK	nd	nd	nd
	p38	nd	nd	nd
	JNK	nd	nd	nd
AI	ERK	170	1.2 × 10^4^	2.0 × 10^−3^
	p38	61	1.6 × 10^4^	9.6 × 10^−4^
	JNK	3	8.4 × 10^4^	534 × 10^4^
p633	ERK	nd	nd	nd
	p38	nd	nd	nd
	JNK	nd	nd	nd

Time‐resolved intensity decays were recorded using time‐correlated single‐photon counting fluorescence lifetime spectrometry (TCSPC FLT) to determine whether phosphorylation by JNK1_α1_ changes the conformation of NOS3. Calmodulin binding to NOS3 produced a change in conformation indicative of active NOS3, observed as a shift to the right, blue to green trace (Fig. S1), as shown previously for NOS1 [32]. Fluorescence decay of JNK1_α1_ phosphorylated NOS3 (orange) did not differ significantly from that of unphosphorylated NOS3 (blue) suggesting little or no change in conformational state. Further, calmodulin produced the same degree of shift (activation) in JNK1_α1_‐treated NOS3, orange to red trace (Fig. S1). The degree of NOS3 phosphorylation by JNK1_α1_ could not be captured; however, these findings differed from our earlier work where ERK2‐treated NOS3 presented an altered conformation from untreated NOS3 [6].

### Endogenous interactions between MAP kinases and NOS3

**Fig. 5:**
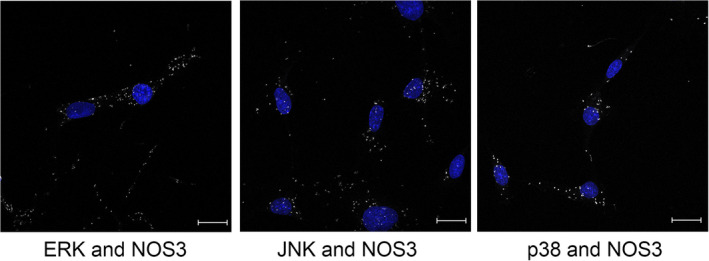
PLA analysis demonstrates that MAP kinases interact with NOS3 *in situ*. HMEC‐1 cells were grown on slides and then subjected to proximity ligation assay. Nuclei shown in blue, interactions between MAP kinases and NOS3 are shown in white, 40× magnification, scale bar = 20 µm.

Immortalized human dermal microvascular endothelial cells (HMEC‐1) were utilized to observe NOS3 interactions with MAP kinases because they retain endothelial characteristics, including expressing detectable levels of endogenous NOS3. Additionally, the treatment of HMEC‐1 cells with EGF or insulin altered NOS3 phosphorylation status (data not shown). All three MAP kinases probed interact with NOS3 in growing cells as shown by PLA (Fig. 5), while antibody‐only or NOS3 alone controls had no signal (data not shown). The nature of PLA does not allow us to interpret differences between the levels of NOS3 interaction with the MAP kinases just that all three clearly interact with NOS3.

## Discussion

The 15 nm *K*_D_ for phosphorylated JNK1_α1_ binding to full‐length NOS3 as determined by BLI is similar to those determined for p38α (80 nm) and ERK2 (160 nm) [4]. This was not surprising since others have qualitatively shown an interaction between NOS3 and JNK [10] and the MAP kinases have similarities in the requirements of binding to activators and substrates [8, 17]. Lack of binding to NOS1 by all three MAP kinases examined thus far strengthens the interpretation that NOS3 is a node for MAP kinase signaling, while NOS1 and NOS2 are not.

ERK2‐, JNK1_α1_‐, or p38α‐mediated phosphorylation of NOS3 did not alter *in vitro* NOS3 activity, while the positive control, PKA increased activity (Fig. 3B). Lack of inhibition differs from our prior results with ERK2, but a different ERK2 (His tagged vs GST) and human NOS3 were used along with an enzymatic assay that is more specific to NO production [6]. We speculate that glutathionylation from contaminating GSH in the SignalChem ERK2 was the likely cause of the inhibition observed previously [6]. Additionally, JNK1_α1_‐mediated phosphorylation (S114) did not meaningfully change the confirmation of NOS3 (Fig. S1), as we observed for ERK2 previously, suggesting that phosphorylation in the AI loop or perhaps glutathionylation from contaminating GSH caused the change in conformation observed with ERK [6].

Each kinase phosphorylated NOS3 differently, and consistent with our prior report using mass spectrometry, ERK2 did not meaningfully phosphorylate S114 when compared to JNK1_α1_ and p38α [6]. Phosphorylation of NOS3 at S114, a site not found in NOS1 or NOS2 (Fig. 1), is catalyzed by a number of kinases, including p38α, JNK1_α1_, and CDKs (Fig. 3A,C) [10, 33, 34]. Phosphorylation at S114 negatively regulates NOS3 activity in cells via indirect mechanisms including blocking c‐Src interactions and recruitment of Pin1 [18, 19].

Like the pentabasic region of the AI loop, the hypothesized JNK binding site we named ‘NtMAP’ is not conserved in NOS1 or NOS2 (Fig. 1), and like the AI site, it has elements that make it capable of interacting with ERK and/or p38, leading us to test both AI and NtMAP independently using peptides [15]. As has been observed for NOS binding to calmodulin [7], p38α, and ERK2 [4], nonideality is present in the fits to one‐state binding models for the peptides. There is clearly a secondary, slower‐on, faster‐off component that could represent biologically relevant complexity such as binding‐induced conformational change; however, it is more likely to represent an artifact such as a misfolded subset of the analyte population. Nevertheless, one‐state models fit very well (see Supplemental Information for goodness‐of‐fit parameters) and explain the vast majority of the binding signal observed and clearly indicate that the unphosphorylated peptides bind the kinases with affinities ranging from low to high nm. Further, the phosphorylated peptides evince almost no affinity for the kinases, thus allowing the conclusion that phosphorylation at S114 or S633 prevents the kinases from binding in those regions.

The presence of phosphate close to basic residues of both binding sites suggests that the phosphates alter surface charge in the binding region, preventing MAP kinase binding. Cargnello and Roux previously suggested that a MSK autophosphorylation site near the D‐domain might be a way to regulate the interaction of MAP kinase binding, though it had not yet been investigated [8]. Herein, we show evidence that phosphorylation regulates MAP kinase binding to NOS3, which could be a more generalizable regulatory mechanism for MAP kinase binding partners. That there are two MAP kinases binding sites on NOS3 could allow more than one kinase to bind NOS3 simultaneously, perhaps up to four given that NOS3 is a dimer.

Our data suggest that JNK1_α1_, ERK2, and p38α compete for the same binding site in the AI loop while the NtMAP site ^96^PRRCLGSLVFPRKL^106^ has higher affinity for JNK1_α1_ and p38α, compared to ERK2 that has relatively lower affinity. The NtMAP site could be a bidirectional D motif site, binding kinases in either the forward direction or the less common reverse direction [16]. This may explain the differential phosphorylation at that site, JNK1_α1_ and p38α may interact in the forward direction phosphorylating S114, while ERK2 may utilize the RK residues C‐terminal to the LXF residues in the reverse direction. This would suggest N‐terminal phosphorylation sites for ERK2 and a candidate would be the S57 site we identified previously [6], though S57 has not yet been observed in cells or *in vivo*. Alternatively, ERK2 and/or p38α may interact with the NtMAP site as a DEF motif (LXFP).

The finding that phosphorylation at the S114 blocks binding suggests the possibility of negative feedback on MAP kinase binding by JNK and p38 phosphorylation. While for the AI site, S633 phosphorylation suggests regulation from other inputs since this site is phosphorylated by other kinases, like PKA [2]. We did some preliminary analyses of a longer peptide that included the S600 site; a phosphate at S600 did not appear to significantly alter binding (data not shown). Altogether our data suggest that regulation of binding by phosphorylation might require a proximal interaction to the basic residues. Intriguingly, in early cellular studies treatment of BAECs with bradykinin caused ERK to disassociate from NOS3 complexes and over time the complex reassociated [9]. Since bradykinin activates PKA which phosphorylates NOS3 at S1177 in addition to S633, it is reasonable to infer that PKA‐mediated phosphorylation plays a role in dissociation of ERK (and other MAP kinases) from NOS3 *in vivo* [35].

## Conclusions

The present results contribute to the growing consensus that MAP kinases regulate NOS3 via direct interactions and phosphorylation. We characterized two unique NOS3 sites that MAP kinases bind with nm affinity but can no longer bind when a phosphate is positioned at a nearby physiologically modified residue. NOS3 regulation via MAP kinase interactions and phosphorylation(s) are likely dependent on cell type and location, the microenvironment, and the landscape of other NOS3 modifications. Our results show that MAP kinases can interact with high affinity at two unique locations that are likely modulated by NOS3 phosphorylation status, providing novel insight into the intersection of these critical pathways.

## Conflict of interests

The authors declare no conflict of interest.

## Author contributions

XKVS, ALC, HQ, LW, BW, KAH, KER, and JCS performed experimental work. XKVS, HQ, BW, JLM, and CAC and interpreted the data. XKVS and ALC wrote initial drafts of the manuscript. JCS performed early experimental work and was part of the inception of the ideas that come to fruition here. CAC designed the work, supervised data collection, analyzed data, and put together the final manuscript. XKVS, ALC, HQ, LW, and JLM edited the manuscript. All authors have given approval to the final version of the manuscript.

## Supporting information


**Fig**. **S1**. Phosphorylation of NOS3 by JNK1α1 evinces no significant change in conformation.
**Table S1**. Global Association‐then‐dissociation model parameters and measures of goodness‐of‐fit.

## Data Availability

The data that support the findings of this study are contained in this article, the supplementary material, or found openly available at the National Center for Biotechnology information (https://www.ncbi.nlm.nih.gov/) accession numbers: 9NP_000611.1, NP_000616.3, and NP_000594.2.
