# Supplementary material for: MAP kinases differentially bind and phosphorylate NOS3 via two unique NOS3 sites
Source: FEBS Open Bio. 2022 Mar 15;12(5):1075–86. doi: 10.1002/2211-5463.13384 (PMC9063426; doi:10.1002/2211-5463.13384)
Supplement: Supplementary file 1 — Fig. S1. Phosphorylation of NOS3 by JNK1α1 evinces no significant change in conformation. Table S1. Global Association‐then‐dissociation model parameters and measures of goodness‐of‐fit. [file FEB4-12-1075-s001.pdf]

Supporting information for MAP kinases differentially bind and phosphorylate NOS3 via two unique NOS3 sites

Supplemental Figure 1:

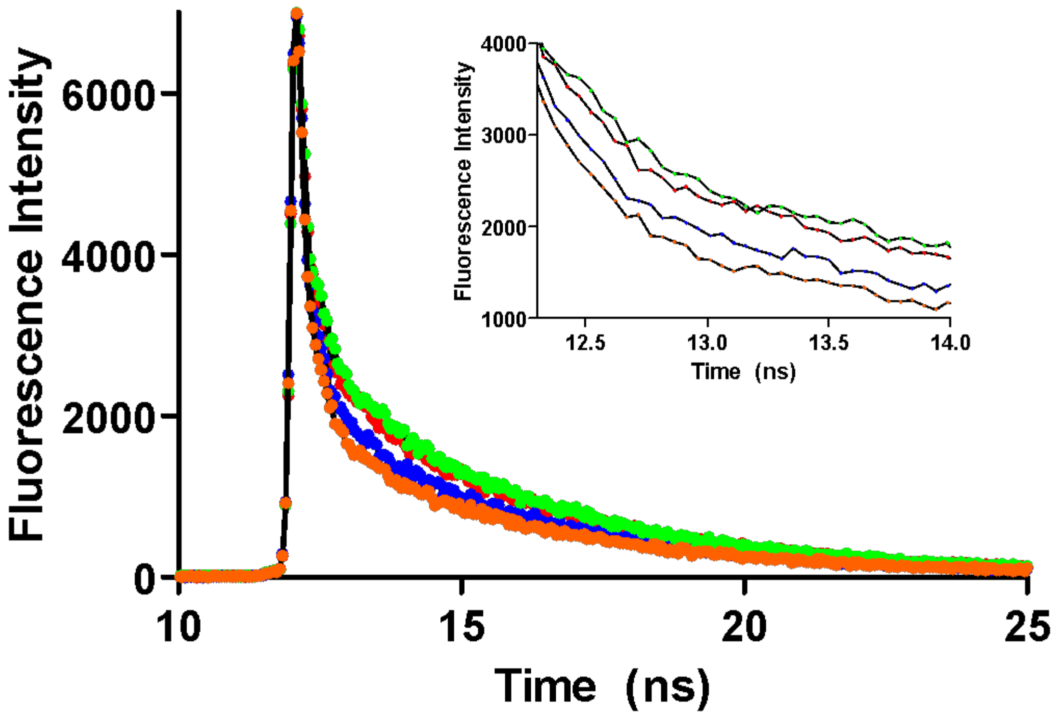

Figure S1. **Phosphorylation of NOS3 by JNK1$_{\alpha 1}$ evinces no significant change in conformation.** Fluorescence decays of NOS3 holoenzyme FMN excited at 473 nm and detected at 525 nm. Blue is NOS3, green is CaM-bound NOS3, orange is JNK1$_{\alpha 1}$-phosphorylated NOS3 and red is JNK1$_{\alpha 1}$-phosphorylated, CaM-bound NOS3.

Supplemental Table 1: Global Association-then-dissociation model parameters and measures of goodness-of-fit

	Fig. 2	Fig. 4 A	B	C	D	E	F
**Best-fit values**							
k_{on} (M-1s-1)	4106	6605	12135	17333	15864	14133	84646
k_{off} (s-1)	0.00006188	0.006226	0.002066	0.0005339	0.0009677	0.0005429	0.0002496
K_D (nM)	15	940	170	31	61	38	3
**95% Confidence Interval**							
k_{on}	4046 to 4165	5914 to 7315	11712 to 12566	16814 to 17864	15210 to 16534	13346 to 14949	83304 to 86002
k_{off}	5.745e-005 to 6.631e-005	0.006059 to 0.006398	0.002021 to 0.002112	0.0005230 to 0.0005449	0.0009290 to 0.001007	0.0005242 to 0.0005620	0.0002362 to 0.0002631
**Goodness of Fit**							
Degrees of Freedom	4498	4498	2246	4498	2248	3748	2248
R squared	0.9987	0.9758	0.9948	0.9883	0.9937	0.9567	0.995
Sum of Squares	4.69	1.488	2.871	5.203	7.294	1.296	11.76
Sy.x	0.03229	0.01819	0.03575	0.03401	0.05696	0.0186	0.07233

Sy.x = standard deviation of the residuals (Y), in nm shift
